# The effect of an interactive weekly text-messaging intervention on retention in prevention of mother-to-child transmission of HIV care: a randomised controlled trial (WelTel PMTCT)

**DOI:** 10.1038/s41598-023-35817-x

**Published:** 2023-06-09

**Authors:** Björn Nordberg, Mia Liisa van der Kop, Winfred Mwangi, Jonathan Mwangi, Eunice Kaguiri, Katrine J. Chamorro de Angeles, Richard T. Lester, Erin E. Gabriel, Susanne Rautiainen, Patricia Opondo Awiti, Anna E. Kågesten, Edwin Were, Anna Mia Ekström

**Affiliations:** 1 Department of Global Public Health, Karolinska Institutet, Tomtebodavägen 18 B, 171 65 Solna, Sweden; 2 Department of Infectious Diseases, Helsingborg Hospital, Helsingborg, Sweden; 3 Directorate of Reproductive Health, Moi Teaching and Referral Hospital, Eldoret, Kenya; 4 Partners in Prevention, Moi University, Eldoret, Kenya; 5 Division of Infectious Diseases, Department of Medicine, University of British Columbia, Vancouver, Canada; 6 Department of Medical Epidemiology and Biostatistics, Karolinska Institutet, Stockholm, Sweden; 7 Clinical Epidemiology Division, Department of Medicine, Karolinska Institutet, Stockholm, Sweden; 8 Department of Reproductive Health, Moi University, Eldoret, Kenya; 9 Department of Infectious Diseases, South General Hospital, Stockholm, Sweden

**Keywords:** Public health, Disease prevention, HIV infections

## Abstract

Retention in prevention of mother-to-child transmission (PMTCT) care is critical to prevent vertical HIV transmission and reduce morbidity and mortality of mother-infant pairs. We investigated whether weekly, interactive text-messaging improved 18-month postpartum retention in PMTCT care. This randomised, two-armed, parallel trial was conducted at six PMTCT clinics in western Kenya. Pregnant women with HIV at least 18 years of age with access to a mobile phone, able to text-message, or had somebody who could text on their behalf, were eligible. Participants were randomly assigned at a 1:1 ratio in block sizes of four to the intervention or control group. The intervention group received weekly text messages asking “How are you?” (“Mambo?” in Swahili) and were requested to respond within 48 h. Healthcare workers called women who indicated a problem or did not respond. The intervention was administered up to 24 months after delivery. Both groups received standard care. The primary outcome was retention in care at 18 months postpartum (i.e., clinic attendance 16–24 months after delivery based on data from patient files, patient registers and Kenya’s National AIDS and STI Control Programme database), which was analysed by intention-to-treat. Researchers and data collectors were masked to group assignment, while healthcare workers were not. Between June 25th, 2015, and July 5th, 2016, we randomly assigned 299 women to the intervention and 301 to standard care only. Follow-up concluded on July 26th, 2019. The proportion of women retained in PMTCT care at 18 months postpartum was not significantly different between the intervention (n = 210/299) and control groups (n = 207/301) (risk ratio 1.02, 95% confidence interval 0.92–1.14, p = 0.697). No adverse events related to the mobile phone intervention were reported. Weekly, interactive text-messaging was not associated with improved retention in PMTCT care at 18 months postpartum or linkage to care up to 30 months postpartum in this setting. (ISRCTN No. 98818734).

## Introduction

Almost a decade after the World Health Organization (WHO) introduced Option B+, a strategy to initiate all pregnant women living with HIV on antiretroviral therapy (ART) for life irrespective of their CD4 cell count^1^, 150 000 children were still infected with HIV in 2020^2^. Most of these cases were attributable to a failure to prevent mother-to-child transmission (PMTCT) of HIV. Before Kenya began to implement Option B+ in 2013, only 40% of women were retained in PMTCT care until the early postpartum period^3^. PMTCT Option B+ comprises antenatal care (ANC), prophylactic ART, infant follow-up including testing, and, ideally, linkage to long-term HIV care. After scaling up Option B+, postpartum retention in care has improved, and 76% of infants in Kenya were brought back for an early infant diagnostic HIV test by 8 weeks of age in 2020^2^. A systematic review conducted in 2020 did, however, still find sub-optimal ART adherence in PMTCT programs in eastern Africa^4^. In 2020, the Kenyan vertical postpartum HIV transmission rate was 5% at 6 weeks postpartum, and at the end of PMTCT care (at 18 months postpartum, or 3 months after the end of breastfeeding if later), it had increased to 10%, demonstrating a continued high risk of HIV transmission during the breastfeeding period^2^. This pattern is similar in several other HIV high-prevalence countries in sub-Saharan Africa and underlines the importance of keeping pregnant women and infants retained until the end of PMTCT care^2^. To eliminate vertical HIV transmission, new interventions are needed to improve adherence to ART and retention among women and infants enrolled in PMTCT care.

Access to mobile phones has increased for women in low- and middle-income countries, including Kenya^5^. This has created opportunities to determine whether text-messaging can be used to improve outcomes in PMTCT care. Before the start of this trial, text-messaging reminders had been found to decrease non-attendance to clinic appointments in various medical disciplines^6^. For people enrolled in general HIV care, mobile phone reminders had shown mixed results on clinic attendance^7,8^, but text-messaging interventions had been found to improve ART adherence^9,10^. So far, randomised controlled trials (RCTs) of text-messaging interventions for women in PMTCT care have shown mixed results^11–16^. Two RCTs from the pre-Option B+ era of pregnant women in Kenya and Mozambique found that text-messaging improved early infant HIV testing^11,12^ and maternal clinic attendance at 8 weeks postpartum^11^. In contrast, two trials conducted in Kenya during^13^ and after^14^ the implementation of Option B+ found that text-messaging did not significantly improve early infant HIV testing^13,14^ or maternal clinic attendance^14^. Until recently, the duration of follow-up in all published trials was short (8-weeks postpartum)^11–14^. In addition, previous trials did not include follow-up telephone calls by healthcare providers in instances where participants did not respond to text-messages, and the frequency and content of the text-messaging interventions differed from the intervention studied here^11–16^.

The WelTel service is an interactive text-messaging check-in designed to improve adherence to ART and retention in HIV care, where weekly text messages asking, “How are you”? (“Mambo?” in Swahili) are sent to participants, who can report problems to get assistance from healthcare workers at their clinic. In contrast to other text-messaging interventions studied in RCTs among women in PMTCT care, the WelTel intervention requires weekly mobile phone interaction. This is intended to increase patients’ engagement in care^17^, and communication between patients and healthcare workers^18^, allowing for the earlier identification of problems, and providing the opportunity for additional assistance or counselling. When the trial began, text-messaging between patients and healthcare workers was not broadly used in western Kenya.

The WelTel intervention improved adherence to ART and HIV viral suppression in a previous RCT involving people living with HIV enrolled in general HIV care in Kenya^9^. A secondary analysis of this trial data suggested that the intervention might have an effect on retention, but that a larger study was needed^19^. As a result, the WelTel Retain trial, conducted between 2013 and 2016, examined retention in care in a general HIV population during the first year of HIV care^20^. The WelTel Retain excluded pregnant women, who may have different motivations for staying in care; therefore, we conducted this trial, WelTel PMTCT, to test whether the intervention could improve retention specifically in a PMTCT population^21^, where improved retention is urgently needed to prevent vertical HIV transmission and reduce morbidity and mortality of mothers and infants. The aim of the WelTel PMTCT trial was to investigate whether a weekly, interactive text-messaging intervention improved 18-months postpartum retention in PMTCT care of women and their new-born infants.

## Methods

### Trial design

WelTel PMTCT was a multi-site, open-label, two-arm, randomised parallel-group trial in which participants were randomly allocated at a 1:1 ratio at six sites in western Kenya.

### Eligibility criteria and recruitment

Pregnant women presenting to one of the study sites with a confirmed HIV diagnosis (either previously known or diagnosed that day) were referred to a research assistant to assess their study eligibility. Pregnant women with confirmed HIV infection and access to a mobile phone who could text message or had someone who could do so on their behalf were eligible for enrolment. Participants were required to be 18 years of age or older, willing to be followed-up for 24 months after delivery, and resident of the PMTCT clinic catchment area with no plans to relocate outside the area during follow-up. Only women attending one of the clinics for their first ANC visit in their current pregnancy were included. The average gestational age at first presentation to ANC is approximately 5 months in Kenya^22^. Women who agreed to participate provided written, informed consent. Those who were illiterate consented by thumb print in the presence of a witness who signed the form. Follow-up included infants born to participants, but only those infants resulting from the pregnancy that made the woman eligible for the trial.

The trial protocol^21^, data collection and informed consent forms were approved by the Moi Teaching and Referral Hospital’s Institutional Research and Ethics Committee (FAN: IREC1292) and was renewed on an annual basis. Ethical approval was also obtained from the Regional Ethics Committee, Stockholm, Sweden (2018/742-31/1). All trial procedures were performed in accordance with relevant guidelines and regulations.

### Study setting

The study took place at six clinical sites in western Kenya that offered antenatal, postnatal, and PMTCT care: Moi Teaching and Referral Hospital; Uasin Gishu District Hospital; Huruma Sub-County Hospital; Kitale County Referral Hospital; Chulaimbo Sub-County Hospital; and Matayos Sub-County Hospital. These clinics provide government-funded public healthcare and are part of the Academic Model Providing Access to Healthcare (AMPATH) program. PMTCT care is free of charge, including ART. Three of the sites are in Eldoret, Kenya’s fifth most populated urban area. Two sites are in smaller towns, and the Kitale County Referral Hospital serves primarily a rural population. Western Kenya has a heterogeneous epidemic of HIV, and the estimated female adult HIV prevalence ranges from 5.5 to 17.4%^23^.

### Intervention

Participants were randomly assigned to receive the WelTel intervention in addition to standard care, or, to standard care only. Every Monday morning, an automated platform sent intervention group participants a text message “Mambo” (Swahili for “How are you?”) Participants were instructed to respond within 48 h of receiving the message either that they were well (“sawa” in Swahili for “okay”) or were having a problem (“shida” in Swahili for “difficulty”). Participants’ responses and instances of non-response were automatically registered in a digital platform.

The WelTel platform automatically forwarded reported problems to a mobile phone at the participant’s local PMTCT clinic where they were followed up by a healthcare worker (peer mentor mother or nurse) who assisted the woman within 48 h. A study coordinator made a list of non-responses every week and delivered it (by paper or text message) to the relevant PMTCT clinic. A healthcare worker telephoned women who did not respond to determine the reason for not responding and to provide assistance if required. If the participant did not answer the call from the healthcare worker, a second call was not made, and the intervention resumed the following week. The weekly messages were sent up to 24 months after delivery, except in the event of a known miscarriage, stillbirth, infant death, maternal death, or participant withdrawal from the intervention, in which case the messages were discontinued.

All participants received Option B+ PMTCT care^1^ and antiretroviral prophylaxis for the infant according to the prevailing guidelines. Standard care included clinical check-ups, CD4 count and viral load monitoring, counselling (HIV, ART adherence, infant feeding), as well as screening for sexually transmitted infections (STI), tuberculosis, and gender-based violence. Women were scheduled to have an appointment 2 weeks after their initial visit and monthly thereafter. Women adherent to ART and virally suppressed had 3-month intervals between visits. A year after delivery, women were scheduled to return to the clinic every 3 months. According to standard practice, clinic staff called all women who missed a scheduled appointment, and those who did not return to the clinic within 1 week of their missed appointment were physically traced.

### Primary and secondary outcomes

The primary outcome was 18-month retention in PMTCT care, measured by whether the mother or infant attended follow-up care between 16 and 24 months after delivery. Follow-up care included clinic appointments, maternal viral load tests, and infant HIV tests. Information about HIV laboratory tests and clinical visits was collected from patient files and registers (antenatal care and HIV-exposed infant registers) at the study sites as well as from the National AIDS and STI Control Programme (NASCOP) (Kenya) database. Visit dates and clinical data were recorded on study-specific forms when the participants were expected to have completed follow-up. NASCOP data were abstracted at the end of follow-up, and data management was completed in September 2020.

Secondary outcomes included: mother or infant retention in PMTCT care between 6 and 12 months postpartum (attending follow-up care 4–14 months after delivery); linkage to chronic HIV care (attending follow-up care 24–30 months after delivery or confirmation that the participant was linked to chronic care); time in care up to 30 months (those who did not have clinical follow-up 24–30 months from enrolment and were not linked to chronic HIV care were considered to have dropped out of care); and facility or home birth. We also assessed the number of infants who were infected with HIV during the trial. Women enrolled in PMTCT care again between 24 and 30 months postpartum (due to a new pregnancy) and women with adverse birth outcomes linked to chronic care before the 24–30 months timeframe were included as being linked to chronic HIV care.

The effect of the intervention on early infant testing of HIV has been reported^24^ and whether the intervention has an effect on maternal viral load will be reported separately.

Before completion of trial, the primary outcome was adjusted to reflect Kenya’s implementation of Option B+, emphasizing mothers’ continuation of ART for life, by expanding the definition of retention from an infant returning for HIV testing at 18–24 months to include evidence of a clinical visit for a mother, or her infant, within 16–24 months postpartum. This allowed women who were still being transferred into chronic HIV care at 18 months postpartum to be considered retained. In addition, we added linkage to chronic HIV care up to 30 months as a secondary outcome to enable a more thorough investigation of long-term retention in PMTCT care. These changes were documented in the ISRCTN registry before any outcome analyses occurred (https://www.isrctn.com/ISRCTN98818734).

### Sample size

Sample size was based on the primary outcome, the proportion of mothers and infants retained in PMTCT care 18 months after delivery, randomised 1:1 to the WelTel intervention group or standard care only. Based on pre-PMTCT Option B+ data available during planning the trial, we expected 30% of participants in the control group to be retained at 24 months^3,25^. With a two-sided α of 0.05 and 80% power, we calculated a sample size of approximately 600 to detect an 11% difference in the primary endpoint (RR 1.37).

### Randomisation

An independent statistician at the Karolinska Institutet who was not involved in enrolling participants generated the randomisation sequence using Stata (Stata Statistical Software: Release 14. College Station, TX: StataCorp LP. 2015). Permuted block sizes of four were used. Randomisation was performed separately at the study sites using sequentially numbered opaque, sealed envelopes. Research assistants assessing trial eligibility did not open the envelope until after the participant was enrolled and had provided consent.

### Blinding

The nature of the intervention precluded blinding of the participants and healthcare workers who responded to the participants’ texts. The laboratory staff and those extracting data from the medical files were blinded, as well as all researchers, until the database was unlocked after the completion of data cleaning.

### Statistical methods

The primary analysis was done on an intention-to-treat basis with all randomly assigned participants. The proportion of mothers or infants retained in care at 18 months postpartum was calculated using Poisson regression with robust standard errors. Sensitivity analyses included adjustment for age (18–24; 25–29; 30–34; 35–44 years), time from HIV diagnosis to enrolment (< 6 months or ≥ 6 months), and site. Additional sensitivity analyses excluded participants with a documented miscarriage, stillbirth, death of the infant or mother, or transfer to another clinic before 18 months postpartum. All adjusted variables and sensitivity analyses were determined a priori.

Unadjusted and adjusted analyses of secondary outcomes were similarly conducted. Retention in care between 6 and 12 months postpartum was analysed using the same exclusion criteria. Logistic regression was used to determine whether there were associations between the intervention and adverse birth outcomes (miscarriage or stillbirth) or transfer to another clinic. We used WHO's definitions of miscarriage and stillbirth, in which babies who die before 28 weeks of pregnancy were considered a miscarriage and those who die at or after 28 weeks were considered stillbirths^26^. For time to drop out of care, we used a Kaplan–Meier analysis and estimated the hazard ratios using Cox models.

After the study protocol had been published, but prior to data analyses, age (18–24; 25–29; 30–34; 35–44 years of age), education (primary or less; secondary; post-secondary), married or living with a partner (yes/no), time from HIV diagnosis to enrolment (< 6 months or ≥ 6 months), HIV status disclosure (yes/no), travel time to clinic (< 1 h or ≥ 1 h) and site, were selected as variables for subgroup analyses. Subgroups were identified based on pre-existing knowledge of barriers to pregnant women’s participation in PMTCT care. Effect modification by subgroup was assessed by introducing each subgroup separately as an interaction term in the unadjusted intervention effect analysis using Poisson regression with robust standard errors. Statistical analyses were done using StataCorp. 2011 and 2017. *Stata Statistical Software: Release 12 and 15*. College Station, TX: StataCorp LP, and StataCorp LLC. The trial and the statistical analysis plan was registered with the ISRCTN registry (ISRCTN98818734, date of first registration: 09/12/2014)^27^.

## Results

**Figure 1:**
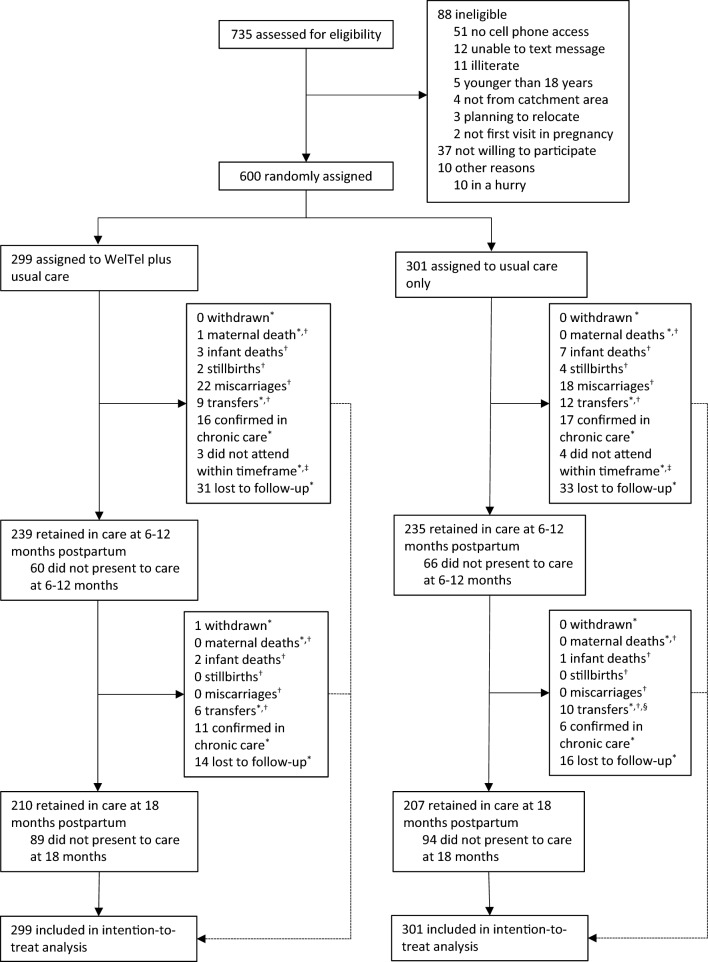
Flow diagram of participants in the WelTel PMTCT trial. *Reasons why participants did not present to care at 6–12 months postpartum or 18-month postpartum outcomes. ^†^Excluded in sensitivity analyses. ^‡^Participants who did not present to care at 6-12 months postpartum but who were retained in care at 18 months postpartum. ^§^10 participants transferred care before the start of the primary outcome timeframe (16–24 months postpartum), one of these participants was retained in care based on data outside of the individual's clinic of enrolment.

Between June 25th, 2015, and July 5th, 2016, 735 women presenting to PMTCT care at one of the six study sites were screened for study eligibility. Of those, 88 were ineligible, the main reason being they lacked cell phone access (Fig. 1). In addition, 37 women were unwilling to participate, and 10 did not have time to be screened. Of 600 women who were eligible and recruited, 299 were randomised to receive the intervention in addition to standard care and 301 to receive standard care only. Follow-up concluded on July 26th, 2019, when the trial ended.

Table 1 presents participants’ baseline characteristics. The mean age was 29.3 years (standard deviation [SD] 5.7) and 81% lived with a partner. Most participants (71%) had been diagnosed with HIV 6 months or earlier (Table 1), and two-thirds were exposed to ART at baseline, the majority of whom (78%) had been taking ART for longer than 6 months. Over 90% of participants used their own mobile phone for the study.

**Table 1:** Baseline characteristics.

	Control (n = 301)	SMS intervention (n = 299)
Age (years)		
18–24	69 (22.9%)	67 (22.4%)
25–29	89 (29.6%)	79 (26.4%)
30–34	83 (27.6%)	83 (27.8%)
35–44	60 (19.9%)	70 (23.4%)
Mean (SD)	29.10 (5.5)	29.55 (5.9)
Education		
Primary or less	129 (42.9%)	147 (49.2%)
Secondary	120 (39.9%)	105 (35.1%)
Post-secondary	52 (17.3%)	47 (15.7%)
Married or living with a partner		
Yes	244 (81.1%)	244 (81.6%)
No	57 (18.9%)	55 (18.4%)
Clinic		
Chulaimbo Hospital or Matayos Hospital	42 (14.0%)	43 (14.4%)
Moi Teaching and Referral Hospital	93 (30.9%)	93 (31.1%)
Kitale County Referral Hospital	103 (34.2%)	102 (34.1%)
Uasin Gishu District Hospital	38 (12.6%)	38 (12.7%)
Huruma Hospital	25 (8.3%)	23 (7.7%)
Travel time to clinic		
< 1 h	208 (69.1%)	214 (71.6%)
≥ 1 h	93 (30.9%)	85 (28.4%)
Time since HIV diagnosis		
< 6 months	84 (27.9%)	93 (31.1%)
≥ 6 months	217 (72.1%)	206 (68.9%)
ART exposed at baseline		
Yes	194 (64.5%)	204 (68.2%)
No	74 (24.6%)	70 (23.4%)
Missing	33 (11.0%)	25 (8.4%)
Time on ART (months)*		
≤ 6	36 (18.6%)	46 (22.5%)
> 6	157 (80.9%)	155 (76.0%)
Missing	1 (0.5%)	3 (1.5%)
HIV-status disclosure		
Yes	232 (77.1%)	226 (75.6%)
No	69 (22.9%)	73 (24.4%)
Mobile phone used for the study		
Own phone	283 (94.0%)	271 (90.6%)
Another’s phone	18 (6.0%)	28 (9.4%)

Data are n (%) or mean (SD).

*SMS* short message service, *SD* standard deviation, *ART* antiretroviral therapy.

*Time on ART is only applicable to those participants who were ART-exposed at baseline (n = 194 in the control group and 204 in the SMS group).

All participants randomised to the intervention and control group were correctly allocated to their respective groups. Participants whose infant had a recorded date of birth received the intervention for a median of 24 months postpartum (IQR 23–24). Of those who were to receive the intervention for the entire 24-month postpartum period, 13 women received messages for shorter time than intended (median 22 months IQR 12–24) for technical or other reasons.

The WelTel platform sent 31,640 text messages to intervention group participants. Two of the 31,640 text messages were sent after a participant had withdrawn, and the responses to those two text messages are therefore not presented. Participants responded “okay” to 15,183 (48.0%) of the text messages and with a problem 438 (1.4%) times. They did not respond to 16,017 (50.6%) of the text messages. During follow-up, 16 participants requested to stop receiving the weekly messages, and one woman in the intervention group withdrew from the study.

**Figure 2:**
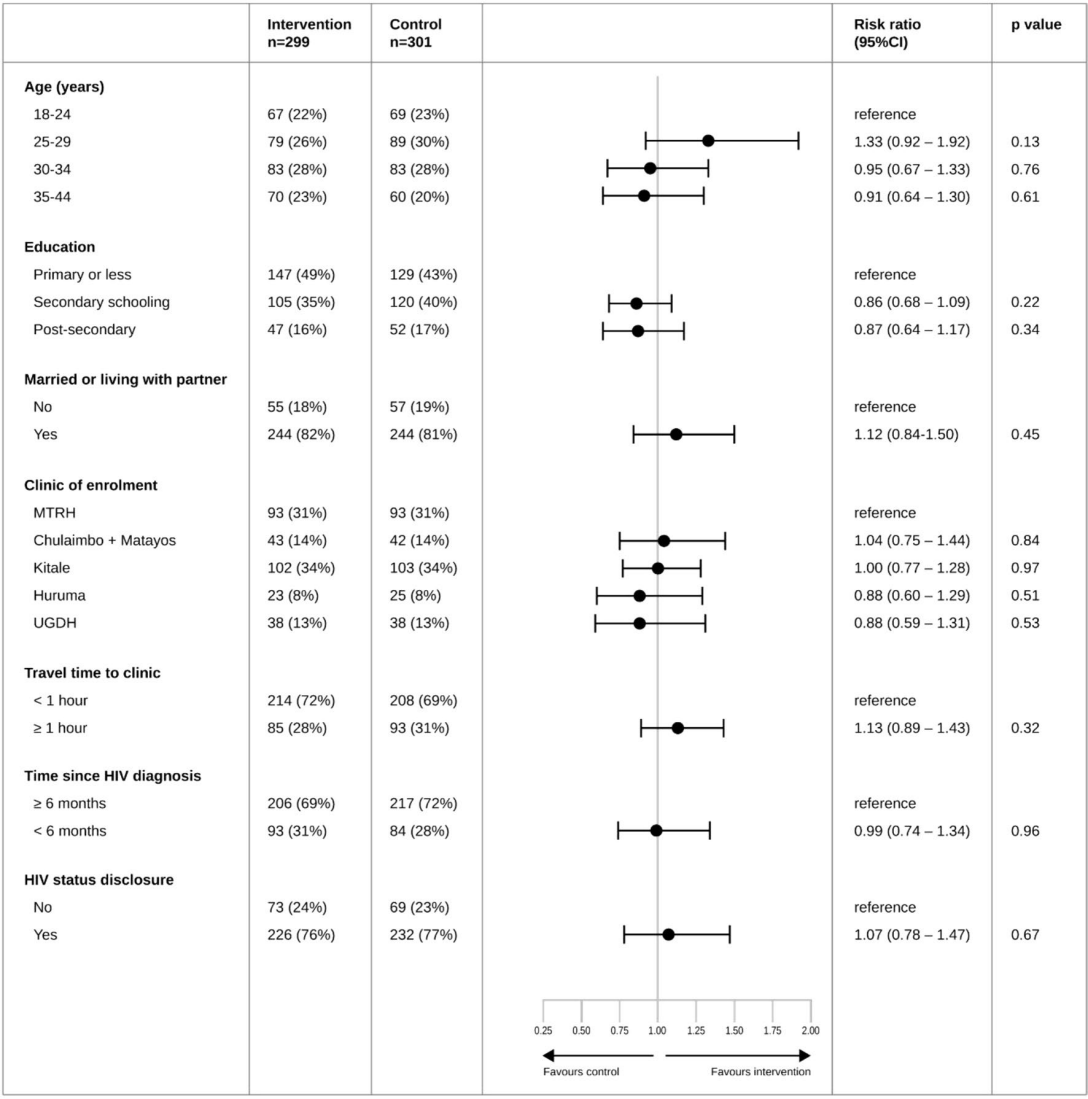
Forest plot of effect modification* across pre-specified subgroups of the effect of the WelTel intervention on 18-months postpartum retention in PMTCT care. *CI* confidence interval, *MTRH* Moi Teaching and Referral Hospital, *PMTCT* prevention of mother-to-child transmission, *UGDH* Uasin Gishu District Hospital. *Effect modification was assessed by introducing each subgroup separately as an interaction term in the unadjusted intervention effect analysis using Poisson regression with robust standard errors.

The intention-to-treat analysis indicated that the intervention was not associated with improved retention in PMTCT care at 18 months postpartum. Retention in care was recorded in 210 of 299 (70.2%) of intervention group participants and 207 of 301 (68.8%) participants in the control group (risk ratio 1.02, 95% confidence interval 0.92–1.14) (Table 2). Adjusting for baseline characteristics and a sensitivity analysis excluding prespecified groups gave similar results. Subgroup analyses, including age, education, marital status, time from HIV diagnosis to enrolment, HIV disclosure, travel time to clinic, and site, indicated no statistically significant differences in the effect of the intervention (Fig. 2). Best- and worst-case scenarios of the intervention's effect on retention in care is presented as supplementary information.

**Table 2:** Primary outcome.

	Intervention n (%)	Control n (%)	Risk ratio (95% CI); p value	Adjusted* risk ratio (95% CI); p value
Retention in care at 18 months postpartum	210/299 (70.2)	207/301 (68.8)	1.02 (0.92–1.14); p = 0.697	1.03 (0.93–1.14); p = 0.582
Retention in care at 18 months postpartum (sensitivity analysis)^†^	208/254 (81.9)	201/248 (81.0)	1.01 (0.93–1.10); p = 0.809	1.02 (0.94–1.10); p = 0.649

*CI* confidence interval.

*Adjusted for baseline characteristics of age, time from diagnosis to enrolment, and site.

^†^Excluded participants with a documented miscarriage; stillbirth; infant death; transfer to another clinic, maternal death before 18 months postpartum.

**Figure 3:**
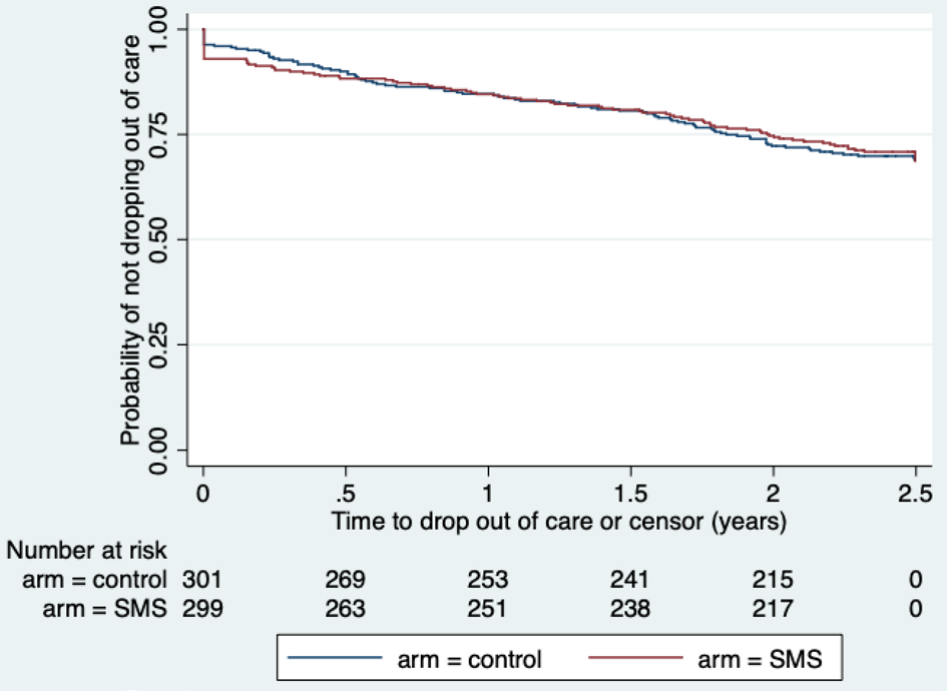
Kaplan–Meier estimate of the survival curve for time to dropping out of care. *SMS* short message service.

Secondary outcomes are presented Table 3. A similar proportion of participants in each group were retained in care between 6 and 12 months postpartum (239 of 299 [79.9%] in the intervention group and 235 of 301 [78.1%] in the control group: risk ratio 1.02, 95% confidence interval 0.94–1.11) (Table 3). Sensitivity analyses, in which we adjusted for age, site, and time from HIV diagnosis to enrolment, supported these results (Table 3). Similarly, the intervention was not associated with a significant increase in linkage to care or giving birth in a facility in either the crude or adjusted analyses. In the intervention group, 89 (29.8%) participants dropped out of care by 30 months from enrolment, compared to 92 (30.6%) participants in the control group, yielding a hazard ratio of 0.98, 95% confidence interval 0.73–1.31 (Fig. 3). Adjusting for baseline characteristics did not change the inference on the association between the WelTel intervention and dropping out of care.

**Table 3:** Secondary outcomes.

	Intervention n (%)	Control n (%)	Risk ratio (95% CI); p value	Adjusted* risk ratio (95% CI); p value
Retention in care between 6 and 12 months postpartum	239/299 (79.93)	235/301 (78.07)	1.02 (0.94–1.11); p = 0.576	1.03 (0.95–1.11); p = 0.499
Retention in care between 6 and 12 months postpartum (sensitivity analysis)^†^	231/258 (89.53)	225/254 (88.58)	1.01 (0.95–1.07); p = 0.730	1.01 (0.95–1.07); p = 0.679
Linkage to chronic care	210/299 (70.23)	209/301 (69.44)	1.01 (0.91–1.12); p = 0.831	1.02 (0.92–1.12); p = 0.737
Facility delivery^‡^	150/299 (50.17)	155/301 (51.50)	0.97 (0.83–1.14); p = 0.745	0.98 (0.84–1.13); p = 0.754

*CI* confidence interval.

*Adjusted for baseline characteristics of age, time from diagnosis to enrolment, and site.

^†^Excluded participants with a documented miscarriage; stillbirth; infant death; transfer to another clinic, maternal death before 14 months postpartum.

^‡^Missing values for 88 participants in the intervention group and 97 in the control group. Outcome not applicable for 48 participants in the intervention group and 42 in the control group due to miscarriage, transfer or lost to follow-up before infant was due. Analysis was intention-to-treat.

During the trial, four infants (1.6%) of 249 recorded live births in the intervention group and three infants (1.2%) of 255 recorded live births in the control group were infected with HIV. The intervention was not associated with any significant adverse birth outcomes or transfer of care (Table 4). No adverse events related to the mobile phone intervention were reported during the trial.

**Table 4:** Adverse birth outcomes and transfer of care.

	Intervention (n = 299)	Control (n = 301)	Odds ratio (95% CI); p value	Adjusted* odds ratio (95% CI); p value
Adverse birth outcomes^†^	24 (8.03%)	22 (7.31%)	1.11 (0.61–2.02); p = 0.741	1.10 (0.60–2.02); p = 0.754
Transfers	15 (5.02%)	22 (7.31%)	0.64 (0.33–1.25); p = 0.190	0.63 (0.32–1.25); p = 0.188

*CI* confidence interval.

*Adjusted for baseline characteristics of age, time from diagnosis to enrolment, and site.

^†^Miscarriage and stillbirth.

## Discussion

To our knowledge, our study is the first RCT in PMTCT care with follow-up up to 30 months postpartum to investigate the effect of a text-messaging intervention. Our trial did not find that this weekly text-messaging intervention improved retention in PMTCT care at 18 months postpartum. Further, we observed no significant effect of the WelTel intervention on retention in PMTCT care between 6 and 12 months postpartum, delivery at a facility, postpartum linkage to chronic HIV care, or dropping out of care by 30 months from enrolment. There was no evidence of an intervention effect among subgroups. After excluding women who had died, had a miscarriage, stillbirth, infant death, or had transferred to another clinic, retention in PMTCT care at 18 months postpartum was 81%, substantially higher than expected in both study groups compared to pre-trial estimates of 30% in the control group^3,25^.

Our findings are consistent with a 2021 three-armed RCT of 824 women in PMTCT care in Kenya that reported no effect of either one-way or two-way text-messaging on loss to follow-up from PMTCT care up to 2 years postpartum^15^. Similarly, a recent cluster-RCT of 1331 women in PMTCT care in Kenya reported no effect of either two-way text-messaging, or two-way text-messaging plus home visits by community-based mentor mothers, on retention in care at 1 year postpartum^16^. Two-way text-messaging in both trials offered women the opportunity to contact their clinic^15,16^, but unlike the WelTel intervention, women were not contacted by healthcare workers for assistance if they did not respond to the text messages.

Our results add to a small but growing body of evidence from RCTs published between 2016 and 2022 that reported no effect of text-messaging interventions on early postpartum (up to 8 weeks)^13,14,24^, 1-year postpartum^16^, and 2-year postpartum retention in PMTCT care^15^. So far, our trial is the only text-messaging RCT that has followed women in PMTCT as they transfer into regular lifelong ART care, i.e., the core idea of WHO’s PMTCT Option B+^1^. Most previous trials have investigated the effect of text-messaging interventions on retention in PMTCT care up to 8 weeks postpartum only and assessed the effectiveness of the intervention on the uptake of early infant HIV testing^11–14^. In a previous paper, we also assessed the impact of weekly text-messaging on early infant HIV testing, a prespecified secondary outcome, but found no significant effect^24^. In contrast, earlier trials from Mozambique and Kenya observed that text-messaging improved the uptake of early infant HIV testing^11,12^ and maternal clinic attendance at 8 weeks postpartum^11^. The discrepancy between older and more recent studies may be due to substantial investments into improving PMTCT care in Kenya and Africa over the past few years, with resulting increased rates of early infant diagnostic HIV testing and decreasing vertical HIV transmission^28^.

We observed that retention in PMTCT care at 18 months postpartum was substantially higher than expected based on data available at the start of this trial across both groups^3,25^. Our estimates are in line with pooled estimates from a 2018 systematic review^29^ and a 2021 RCT from Kenya^15^. Improved retention in PMTCT care is likely due to several factors. The introduction and implementation of PMTCT Option B+ has been described as a game-changer. Simpler treatment programs, improved quality of care and access to ART, and earlier treatment initiation has improved health outcomes for women and reduced the risk of HIV transmission to infants and sexual partners^30^. In addition, several nation-wide interventions were also implemented to improve retention in PMTCT care in Kenya^31–34^. Important interventions were also integrated in standard care in our particular study setting between 2005 and 2017: tracing women who defaulted on scheduled appointments by phone or household visit^31,33^; peer-support and counselling by mentor mothers^32^; and, importantly, an integration of general antenatal, postnatal, and PMTCT care took place to reduce the stigma related to visiting clinics exclusively for people living with HIV^32,34^.

Mobile phone access was high in our study setting. Only 7% of pregnant women screened were excluded due to a lack of mobile phone access. In 2015, coinciding with the beginning of our trial, Kenya launched new guidelines for ART providers, recommending increased use of telecommunication to improve adherence to clinic visits and ART^31^. This likely contributed to the increased use of telecommunication as part of standard care in Kenyan HIV and PMTCT care and at our study sites during the trial. In qualitative interviews with healthcare workers and participants during our trial, we found that informal phone communication between healthcare workers and patients took place in both groups^35^. This included healthcare workers using their private phones and contacting patients outside of the clinic’s operating hours^35^. In addition, increased use of telephone calls and text-messaging to deliver laboratory test results, appointment reminders, and to guide and refer patients with questions or problems were part of programmatic strategies implemented for all women in PMTCT care. Altogether, important changes in care provision, increased use of telecommunication, and general improvements in retention in care may have decreased any potential added value of the WelTel service and attenuated any effect of the intervention on retention in care.

At the end of 2019 when follow-up was completed, 98% of Kenyans had a mobile connection^36^. There was a corresponding increase in Kenyans’ use of social media, averaging over 3 hours per day in the 21–35 years age group in 2018^37^. It is possible that this rapid increase in text-messaging and social media use could reduce the impact of a weekly message. Previous RCTs from Kenya also reported increased mobile phone communication^13^ and the introduction of programmatic support mechanisms as part of standard care^14^ as possible reasons for not finding an effect of text-messaging on early infant HIV testing. Increased mobile phone use creates new opportunities for health communication, but also requires more innovative and flexible mobile health (mHealth) interventions to penetrate this massive influx of information. New mHealth interventions need to be considered within the context of any current cellphone-based communication already in place in the health care setting. Rigorous evaluation of mobile health interventions is also required. There are many studies of mHealth applications in a broad range of conditions; however, the characteristics of text-messaging interventions, such as frequency, interactivity, timing, personalization, and the integration of advanced messaging capabilities with smart phones, are an understudied area. Critical evaluation is needed to efficiently advance the field and ensure that we spend health resources wisely.

Less discrimination towards people living with HIV in Kenya^28^ may also have contributed to improved uptake and retention in PMTCT care in recent years. Women in our trial reported a relatively low prevalence of social concerns related to PMTCT participation^38^, a finding supported by a 2017 study from Uganda^39^. When WelTel was studied to examine retention in (non-PMTCT) HIV care in Kenya, there was a significant finding of improved quality of life among those who received the text-message intervention^20^, but this was not assessed in our trial.

To our knowledge, this is the first RCT that has investigated whether a text-messaging intervention improved long-term retention including women’s postpartum linkage to chronic HIV care. Linkage to chronic HIV care with sustained ART to ensure viral suppression is an important goal of the Option B+ recommendations to prevent HIV disease progression among postpartum women and sexual transmission of HIV to partners^30^. Only 70% in the intervention group and 69% in the control group were linked to chronic HIV care at the end of PMTCT care, which indicates that more effort is needed to reach the UNAIDS’ target of sustained ART with viral suppression among 95% of all people living with HIV^40^. Our study also had a much lower proportion of infants with a recorded live birth who were infected with HIV (1.4%), compared to the national Kenyan vertical transmission rate of 10% in 2020^2^. The proportion of infants infected with HIV in our trial did however not include transmission from women who were unaware of a positive HIV status or did not access ART.

A key strength of this trial is its long duration of follow-up, from the first appointment in antenatal care up to 30 months postpartum. In addition, the participation rate was high and included both rural and urban study sites spread over a large area in western Kenya, contributing to the generalisability of our results. We designed the intervention to resemble real-life conditions, with no incentives given to the participants. In addition, we used multiple sources of data including patient files, patient registers, and a national database to maximise the validity of our outcomes. Using a national database improved the accuracy of our retention in care estimate compared to using clinical records alone, since women who had a viral load test outside of their study clinic were also captured.

Trial limitations include the open-label design necessitating healthcare workers’ awareness of the intervention to call women who reported a problem. Despite instructions to healthcare workers not to implement the components of the intervention in the control group, increased mobile phone communication with clients in general was reported by both clinic staff and peer mothers in our post-trial facility evaluation interviews (data unpublished), possibly improving retention for all study participants, undermining the possibility to observe an effect of the intervention. We were unable to quantitatively assess the frequency of all types of tele-communication between participants and healthcare providers. There was, however, no report of WelTel text messages sent to participants in the control group. The overall increase in mobile phone use was most likely due to national and local initiatives to improve HIV and PMTCT care outcomes^31,33^. Participation in a research study may influence participants’ behaviour through the Hawthorne effect and improve retention in care in the control group more than for women in PMTCT care who are not participating in a research study.

In 2012, the WHO recommended text-messaging and more efficient patient tracking to improve retention throughout the continuum of care for people living with HIV^41^. Our results do not contradict this recommendation but suggest that today, weekly interactive text-messaging may not further improve retention in care in settings that already have introduced multiple support mechanisms, such as western Kenya. Thus, our results cannot be generalised to settings without similar support mechanisms or with greater barriers to uptake and retention in care, where the intervention may provide added value. A potential benefit of using health messaging platforms to text patients rather than using healthcare providers’ private phones, is improved documentation for health records and capturing program-level data that can be used for quality improvement.

## Conclusion

In conclusion, we did not find that weekly text-messaging improved 18-month postpartum retention in PMTCT care or linkage to care up to 30 months postpartum. Our results and those of other studies suggest improved retention throughout PMTCT care is likely due to an overall increase in much-needed support mechanisms as part of standard HIV care in Kenya, including the use of both formal and informal telecommunication between patients and healthcare workers.

## Supplementary Information

Supplementary Information.

## Data Availability

The datasets generated during and/or analysed during the current study are available from the corresponding author on reasonable request.

## References

[1] World Health Organization. Programmatic update: Use of antiretroviral drugs for treating pregnant women and preventing HIV infection in infants: Executive summary. https://apps.who.int/iris/handle/10665/70892 (2012). Accessed 10 March 2022.

[2] UNAIDS. Start Free, Stay Free, AIDS Free—Final report on 2020 targets. https://www.unaids.org/en/resources/documents/2021/start-free-stay-free-aids-free-final-report-on-2020-targets (2021). Accessed 11 April 2022.

[3] Azcoaga-Lorenzo A (2011). Effectiveness of a PMTCT programme in rural Western Kenya. AIDS Care.

[4] Tsegaye R (2020). The magnitude of adherence to option B plus program and associated factors among women in eastern African countries: A systematic review and meta-analysis. BMC Public Health.

[5] GSM Association. Connected Women—The Mobile Gender Gap Report 2019. https://www.gsma.com/mobilefordevelopment/wp-content/uploads/2019/02/GSMA-The-Mobile-Gender-Gap-Report-2019.pdf (2019). Accessed 11 April 2022.

[6] Downer SR, Meara JG, Da Costa AC (2005). Use of SMS text messaging to improve outpatient attendance. Med. J. Aust..

[7] Kliner M, Knight A, Mamvura C, Wright J, Walley J (2013). Using no-cost mobile phone reminders to improve attendance for HIV test results: A pilot study in rural Swaziland. Infect. Dis. Poverty..

[8] Kunutsor S (2010). Using mobile phones to improve clinic attendance amongst an antiretroviral treatment cohort in rural Uganda: a cross-sectional and prospective study. AIDS Behav..

[9] Lester RT (2010). Effects of a mobile phone short message service on antiretroviral treatment adherence in Kenya (WelTel Kenya1): A randomised trial. Lancet.

[10] Pop-Eleches C (2011). Mobile phone technologies improve adherence to antiretroviral treatment in a resource-limited setting: A randomized controlled trial of text message reminders. AIDS (London, England)..

[11] Odeny TA (2014). Texting improves testing: A randomized trial of two-way SMS to increase postpartum prevention of mother-to-child transmission retention and infant HIV testing. AIDS (London, England)..

[12] Joseph-Davey, D. P.W., Augusto, O., Traca, D., Jetha, E., De Palha, C. Improved uptake of institutional birth and early infant HIV testing following SMS reminders among PMTCT patients in Mozambique. In *7th IAS conference on HIV pathogenesis, treatment and prevention*, Jun 30-Jul 3, Kuala Lumpur, Malaysia (2013).

[13] Kassaye SG (2016). Cluster-randomized controlled study of SMS text messages for prevention of mother-to-child transmission of HIV in rural Kenya. AIDS Res. Treat..

[14] Odeny TA (2019). Text messaging for maternal and infant retention in prevention of mother-to-child HIV transmission services: A pragmatic stepped-wedge cluster-randomized trial in Kenya. PLoS Med..

[15] Kinuthia J (2021). SMS messaging to improve retention and viral suppression in prevention of mother-to-child HIV transmission (PMTCT) programs in Kenya: A 3-arm randomized clinical trial. PLoS Med..

[16] Abuogi LL (2022). Effects of behavioural interventions on postpartum retention and adherence among women with HIV on lifelong ART: The results of a cluster randomized trial in Kenya (the MOTIVATE trial). J. Int. AIDS Soc..

[17] Smillie K (2014). A qualitative study investigating the use of a mobile phone short message service designed to improve HIV adherence and retention in care in Canada (WelTel BC1). J. Assoc. Nurses AIDS Care JANAC..

[18] Smillie K (2014). Mobile health for early retention in HIV care: A qualitative study in Kenya (WelTel Retain). Afr. J. AIDS Res. AJAR..

[19] van der Kop ML (2014). Factors associated with attrition from HIV care during the first year after antiretroviral therapy initiation in Kenya. J. AIDS Clin. Res..

[20] van der Kop ML (2018). Effect of an interactive text-messaging service on patient retention during the first year of HIV care in Kenya (WelTel Retain): An open-label, randomised parallel-group study. Lancet Public Health..

[21] Awiti PO (2016). The effect of an interactive weekly mobile phone messaging on retention in prevention of mother to child transmission (PMTCT) of HIV program: Study protocol for a randomized controlled trial (WELTEL PMTCT). BMC Med. Inform. Decis. Mak..

[22] Magadi MA, Madise NJ, Rodrigues RN (2000). Frequency and timing of antenatal care in Kenya: Explaining the variations between women of different communities. Social Sci. Med. (1982).

[23] Ministry of health. Kenya HIV Estimates Report 2018. https://nacc.or.ke/wp-content/uploads/2018/11/HIV-estimates-report-Kenya-20182.pdf (2018). Accessed 10 February 2022.

[24] Nordberg B (2021). The effect of weekly interactive text-messaging on early infant HIV testing in Kenya: A randomised controlled trial (WelTel PMTCT). Sci. Rep..

[25] Hassan AS (2012). Dynamics and constraints of early infant diagnosis of HIV infection in Rural Kenya. AIDS Behav..

[26] World Health Organization. Maternal and perinatal death surveillance and response: Materials to support implementation. Geneva. https://www.who.int/publications/i/item/9789240036666 (2021). Accessed 19 April 2023.

[27] Ekstrom AM (2014). The effect of weekly text messaging to improve retention across the prevention of mother to child transmission of HIV program. ISRCTN..

[28] UNAIDS. UNAIDS data 2020. https://www.unaids.org/en/resources/documents/2020/unaids-data (2020). Accessed 11 April 2022.

[29] Knettel BA (2018). Retention in HIV care during pregnancy and the postpartum period in the option B+ era: Systematic review and meta-analysis of studies in Africa. J. Acquired Immune Deficiency Syndromes (1999)..

[30] UNAIDS. On the Fast-Track to an AIDS-Free Generation. https://www.unaids.org/en/resources/documents/2016/GlobalPlan2016 (2016). Accessed 11 April 2022.

[31] Ministry of health. Job Aids for ART Providers in Kenya. http://guidelines.health.go.ke:8000/media/JOBAIDS_for_ART_providers_in_Kenya_February_2015.pdf (2015). Accessed 10 February 2022.

[32] Ministry of health. Kenya framework for elimination of mother-to-child transmission of HIV and Syphilis 2016–2021. https://www.nascop.or.ke/prevention-of-mother-to-child-transmission-downloads/ (2015). Accessed 10 February 2022.

[33] Rachlis B (2015). Implementation and operational research: evaluating outcomes of patients lost to follow-up in a large comprehensive care treatment program in western Kenya. J. Acquired Immune Deficiency Syndromes (1999)..

[34] Berlacher M (2021). Integrating prevention of mother-to-child transmission of HIV care into general maternal child health care in Western Kenya. Int. J. MCH AIDS..

[35] ChamorroDeAngeles KJ (2022). Care from distance, yet closer together: How an SMS intervention enhanced care engagement for prevention of mother-to-child HIV transmission care in Western Kenya. SSM Qualit. Res. Health..

[36] DataReportal. Digital 2020: Kenya. https://datareportal.com/reports/digital-2020-kenya; (2020). Accessed 11 April 2022.

[37] USIU Africa SIMElab. Social Media Consumtion in Kenya: Trends and Practices. https://www.usiu.ac.ke/assets/file/SIMElab_Social_Media_Consumption_in_Kenya_report.pdf (2019). Accessed 11 April 2022.

[38] Nordberg B (2020). Social concerns related to HIV status disclosure and participation in the prevention of mother-to-child transmission of HIV care among pregnant women in Kenya. BMC Pregnancy Childbirth.

[39] Naigino R (2017). HIV status disclosure and associated outcomes among pregnant women enrolled in antiretroviral therapy in Uganda: A mixed methods study. Reprod. Health.

[40] UNAIDS. Fast-Track - Ending the AIDS epidemic by 2030. https://www.unaids.org/en/resources/documents/2014/JC2686_WAD2014report (2014). Accessed 11 April 2022.

[41] World Health Organization. Retention in HIV programmes: defining the challenges and identifying solutions: meeting report, 13–15 September 2011. https://apps.who.int/iris/handle/10665/44878 (2012).

