# Supplementary material for: The effect of an interactive weekly text-messaging intervention on retention in prevention of mother-to-child transmission of HIV care: a randomised controlled trial (WelTel PMTCT)
Source: Sci Rep. 2023 Jun 9;13:9389. doi: 10.1038/s41598-023-35817-x (PMC10256764; doi:10.1038/s41598-023-35817-x)
Supplement: Supplementary file 1 — Supplementary Information. [file 41598_2023_35817_MOESM1_ESM.docx]

**Supplementary material**

For the primary outcome, we estimated best- and worst-case scenarios for the intervention. The best-case scenario assumed that all those in the intervention group who were excluded in sensitivity analyses due to miscarriage, stillbirth, death of the infant or mother, or transfer to another clinic before 18 months postpartum and *not* retained in care were retained in care, while all those excluded from the control group and not retained in care were assumed to be lost to follow-up. The worst-case scenario, the opposite of this, was also computed. Both scenarios were adjusted for age, time since diagnosis, and site.

**Supplementary table.** Best and worst cases scenarios for the primary outcome.

|  | **Intervention (n=299)** | **Control (n=301)** | **Risk ratio (95% CI); p value** |
| --- | --- | --- | --- |
| Best-case scenario^*, †^  Retention in care at 18 months postpartum | 254 (84.95%) | 207 (68.77%) | 1.24 (1.14-1.35); p<0.001 |
| Worst-case scenario^*, ‡^  Retention in care at 18 months postpartum | 210 (70.23%) | 254 (84.39%) | 0.84 (0.77 – 0.91); p<0.001 |

CI=confidence interval.

*adjusted for baseline characteristics of age, time from diagnosis to enrolment, and site.

^†^best case: assuming all those excluded (participants with a documented miscarriage; stillbirth; infant death; transfer to another clinic, maternal death before 18 months postpartum) from the intervention group and not retained in care *were* retained in care and those excluded from the control group and not retained in care as not retained in care

^‡^worst case: assuming all those excluded (participants with a documented miscarriage; stillbirth; infant death; transfer to another clinic, maternal death before 18 months postpartum) from the intervention group and not retained in care *were* not retained in care and those excluded from the control group and not retained in care as retained in care
